# Macrophage-Derived Extracellular Vesicles: A Promising Tool for Personalized Cancer Therapy

**DOI:** 10.3390/biomedicines10061252

**Published:** 2022-05-27

**Authors:** Antonella Barone, Nicola d’Avanzo, Maria Chiara Cristiano, Donatella Paolino, Massimo Fresta

**Affiliations:** 1 Department of Experimental and Clinical Medicine, University “Magna Græcia” of Catanzaro Campus Universitario-Germaneto, Viale Europa, 88100 Catanzaro, Italy; barone@unicz.it (A.B.); mchiara.cristiano@unicz.it (M.C.C.); 2 Department of Pharmacy, University “G. d’Annunzio” of Chieti-Pescara, Via dei Vestini n.31, 66100 Chieti, Italy; nicola.davanzo@unich.it; 3 Department of Health Science, University “Magna Græcia” of Catanzaro Campus Universitario-Germaneto, Viale Europa, 88100 Catanzaro, Italy; fresta@unicz.it

**Keywords:** macrophages, extracellular vesicles, cancer therapy, personalized nanomedicine, drug delivery

## Abstract

The incidence of cancer is increasing dramatically, affecting all ages of the population and reaching an ever higher worldwide mortality rate. The lack of therapies’ efficacy is due to several factors such as a delay in diagnosis, tumor regrowth after surgical resection and the occurrence of multidrug resistance (MDR). Tumor-associated immune cells and the tumor microenvironment (TME) deeply affect the tumor’s progression, leading to several physicochemical changes compared to physiological conditions. In this scenario, macrophages play a crucial role, participating both in tumor suppression or progression based on the polarization of onco-suppressive M1 or pro-oncogenic M2 phenotypes. Moreover, much evidence supports the pivotal role of macrophage-derived extracellular vesicles (EVs) as mediators in TME, because of their ability to shuttle the cell–cell and organ–cell communications, by delivering nucleic acids and proteins. EVs are lipid-based nanosystems with a broad size range distribution, which reflect a similar composition of native parent cells, thus providing a natural selectivity towards target sites. In this review, we discuss the impact of macrophage-derived EVs in the cancer’s fate as well as their potential implications for the development of personalized anticancer nanomedicine.

## 1. Introduction

Despite the ever increasing efficacy of anticancer therapies, the worldwide mortality rate increases as well [1], reaching around 10 million cancer deaths in 2020 [2]. Several factors such as tumor relapse, unsuitable physicochemical characteristics of the anticancer drugs and related poor pharmacokinetic profiles can affect the fate of the conventional treatments, leading to inefficacious cares [3]. Among these drawbacks, multidrug resistance (MDR) is one of the main factors that leads to the lack of therapies’ efficacy, by reducing the responsiveness of the cancer to the common chemotherapeutic agents as a result of the efflux pumps’ overexpression, the hypoxia condition of the tumor microenvironment (TME), epigenetic modifications, and cancer stem cells’ implication [4,5,6,7]. 

In this context, the TME and, in particular, immune cells associated with it play a crucial role in cancer suppression/progression, deeply influencing the tumor behavior [8]. Macrophages are a class of the immune system’s cells and are placed in all tissues, including in the TME, taking the name of tumor-associated macrophages (TAMs). Considering this close connection between TAMs and cancer, they become clinically relevant for the treatment of solid tumors [9,10,11]. TAMs show two different kinds of phenotype: M1 and M2 [12]. The M1 phenotype is often obtained by the activation of several proinflammatory factors such as bacterial lipopolysaccharides (LPS), interferon gamma (INF-γ) and others. TME is strongly associated with the inflammatory state, and for these reasons, the M1 phenotype macrophages classically provide tumor suppressive activity [13,14]. Due to their plastic behavior, the M1 phenotype can be shifted to an M2 one, thus promoting tumor progression by the secretion of chemokines and cytokines that contribute to the suppression of the inflammatory response [15]. 

Among several approaches carried out both to favor the oncosuppressive phenotype polarization and target TAMs specifically [16], the nanotechnological approach has proposed a heterogeneous nanoplatform composed of different drug delivery systems able to interact directly with TAMs and/or exploit their features with the aim of realizing efficacious anticancer therapies [17,18,19]. 

In this regard, extracellular vesicles (EVs) are known as vesicular messengers into the body by delivering bioactive payloads both in physiological and pathological states, such as cancer [20]. They contribute to the crosstalk between cancer cells and their surroundings, participating in different steps from cell proliferation and migration up to metastasis formation [21]. Recently, immune system-derived EVs have gained attention because of their role into the TME, capable of modulating both the phenotype and the function of the recipient cells. Briefly, macrophage-derived EVs reflect the immunomodulatory features of the donor cells. For this reason M1-derived EVs can induce a therapeutic immune response towards cancer [22]. Their peculiar role in the immune response makes them suitable also for the treatment of different diseases such as autoimmune, neurodegenerative and infectious pathologies, in the form of vaccines for this last one [23]. 

On these bases, the aim of this review is to deeply investigate the role of the macrophage-derived extracellular vesicles as a potential anticancer treatment. Furthermore, the functionalization of these macrophage-derived EVs was also taken into account in order to provide a broader perspective about the development reached in this recent field. 

## 2. Role of Tumor Microenvironment in Cancer Progression

During the last two decades, ever increasing evidences have highlighted the pivotal role of the TME in cancer progression and prognosis [24,25,26]. In fact, it is well-established that solid tumors develop a dynamic and heterogeneous environment that is made up by cancer cells, stromal cells, different infiltrating immune-cells and an extracellular matrix (ECM) [27]. 

In particular, fibroblasts are the most abundant stromal cells in the TME, where they are recognized by the peculiar name of cancer-associated fibroblasts (CAFs). These cells are morphologically similar to the physiologic fibroblasts, but in comparison to these, CAFs are over-activated and do not undergo apoptotic processes [28]. CAFs actively contribute to tumor progression by providing physical support to cancer cells (in association with ECM) and releasing several factors, such as VEGF and FGF, that stimulate the angiogenesis [29]. These cells also play a crucial role during the metastatic processes by the activation of several pathways, including the release of different enzymes able to induce ECM degradation, and then improving cellular migration and invasiveness [30,31]. The ECM is a “cell-derived scaffold” and is a pivotal physical support for all tissues in the human body (except for circulating cells) [32]. It is composed by a series of macromolecules, such as elastin, hyaluronic acid, polysaccharides, proteoglycans, growth factors, etc., and, in association with CAFs, form tumor stroma in solid cancers [33]. The physical properties of the ECM, such as density, porosity and stiffness, strongly reflect its composition and are dramatically altered in the TME. In fact, during the early stage of tumor development, the over-crosslinked collagen leads to an improved physical support for tumor growth, reducing, at the same time, the passage of chemotherapeutic agents, while ECM degradation during the late stages of cancer development improves the spread of metastases [28,34]. In this scenario, several therapeutic approaches have been proposed to remodel the ECM, although its double-edged sword makes it hard to develop therapies which are able to by-pass this physical barrier without inducing excessive modifications in its supramolecular structure, which may result in the metastases spreading through the body [35,36].

A great attention has been also directed to the infiltrating immune-cells and their continuous and dynamic interaction with cancer cells [37,38,39]. Indeed, despite the primary and physiological antitumor effect carried out by this subset of cells during the early stages of cancer development, the pathological physicochemical stimuli in the TME, such as up-regulated growth factors, chemokines, dis-regulated enzyme pathways, etc., may lead to several changes in cancer-associated immune-cells, reversing their function from tumor-suppressive to pro-oncogenic [40]. In these attempts, several monoclonal antibodies able to induce tumor T cells’ response have been approved by the FDA for the treatment of different cancers, such as small lung cancer and melanoma [41]. 

Apart from the cellular involvement in the TME, other altered physical stimuli affect tumor development, as well as the changes that occur in the TME during the cancer progression. In this scenario, the main physical stimuli involved are hypoxia and acidosis [42]. The rapid growth rate of cancer leads to the formation of hypoxic areas that stimulate a huge release of angiogenic mediators, such as VEGF, thus resulting in the neovascularization of the TME. However, because of the immature behavior of these cells, the new vasculature is leaky and shows a poor perfusion efficiency [28]. In these attempts, the oxygen level in solid tumors, especially in the central necrotic area, is lower than physiological conditions. This alteration leads to the activation of several compensative pathways that alter the physiological behavior of cancer cells and further modify the surrounding TME by the modification of cytokines and growth factors’ release. In particular, the activation of the hypoxia-induced factor (HIF) alters the cells’ death mechanisms and promotes the cancer cells’ survival by inhibiting apoptotic processes [43]. This factor is also strongly implicated in the tumor’s metabolic changes by inducing the overexpression of trans-membrane glucose transporters, exacerbating the Warburg effect and then promoting glycolysis instead of the oxidative phosphorylation of glucose [43,44]. This glucose metabolic alteration, in turn, leads to a large production of lactate which is accumulated in the extracellular microenvironment and leads to the tumor acidosis. Together, hypoxia and acidosis induce the activation of several genes, such as pyruvate kinase M2, lactate dehydrogenase A, etc., that further alter the TME and the cancer development [44,45,46]. Hypoxia and acidosis also directly contribute to the occurrence of MDR phenomena. Indeed, the hypoxic environment reduces the capability of chemotherapeutic drugs to reach the deepest area of the tumor (leakage vasculature) and limits the efficacy of radiotherapies because of a limited production of ROS [47]. On the other hand, acidosis strongly inhibits the uptake and/or the efficacy of weak acids and weak bases [48]. Moreover, the high amount of lactate in the TME may also induce several metabolic and epigenetic alterations, modifying the immune response through the induction of macrophage M2-polarization and/or triggering the differentiation of T helper 1 cells [49,50,51]. 

**Figure 1:**
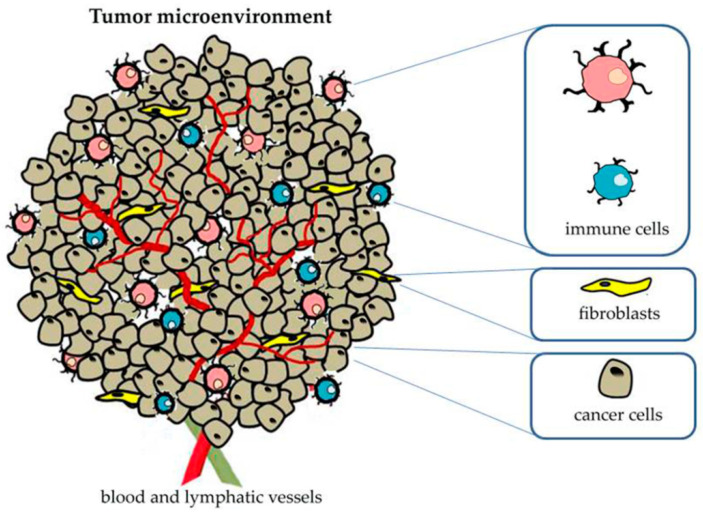
Schematic representation of tumor microenvironment and main associated components.

All the above mentioned components influence each other through bidirectional communication processes that affect cancer development and the responsiveness to the therapies, and make the development of new anticancer treatments which are able to take into account the complex nature of the TME a priority [52,53,54,55] (Figure 1).

### Tumor-Associated Macrophages

Macrophages can be classified into two main distinct classes based on their origin: macrophages derived from circulating monocytes after extravasation into inflamed tissues and tissue-resident macrophages [56,57]. The first kinds of macrophages differentiate themselves during the inflammatory affections. In fact, the abnormal amount of chemokines produced during this pathological alteration, such as IFN-γ, the macrophage colony-stimulating factor (M-CSF), CCL2, CCL5, etc., induced a strong chemo-attraction of circulating monocytes, and after extravasation, they differentiated into macrophages [58,59]. On the other hand, tissue-resident macrophages exist in quite all tissues, e.g., in the liver (Kupffer cells), lung (alveolar macrophages), kidneys (mesangial cells), brain (microglial cells), etc., and contribute actively to the maintenance of homeostasis conditions [60].

Due to the chronic inflammation observed in the tumor area, a large amount of monocytes and then differentiated macrophages have been observed in the TME. They are usually recognized as TAMs and can reach up to 50% of tumor mass [61,62]. TAMs can show a different localization in the TME, such as the fibrous tissue of the tumor stroma or deep tumor nests [63].

These cells can show different behaviors and functions based on their phenotype polarization. In other words, macrophages can follow two different polarization routes, M1 and M2, although this classification only indicated the two potential extremes without taking into account all the intermediated sub-types. M1-like macrophages (also known as classical activated macrophages) are strongly associated with the early stage of tumor development and show huge antitumorigenic effects by the production of a wide range of pro-inflammatory cytokines (e.g., IL-12, IL-23, etc.) and by the release of cytotoxic mediators, such as nitric oxide (NO) and reactive oxygen species (ROS). On the other hand, M2-like macrophages (also known as alternative activated macrophages) are massively present in tumor mass during the late stages of tumor progression and are usually associated with a poor patient prognosis [64]. The M2-like macrophages’ polarization is due to several modifications that occur in the TME and is mainly associated with the release of specific cytokines that trigger a cascade reaction, which leads to a strong immunosuppression. In particular, tumor cells release a large amount of TGF-β and IL-4 that rapidly pushes down the release of IL-12 from the macrophages, thus reducing the activation of T cells and the recruitment of natural killer (NK) cells [65]. At the same time, the release of other peculiar cytokines from cancer necrotic cells, such as IL-10, as well as the presence of other M2-phenotype inducer agents, e.g., vitamin D3, glucocorticoids, etc., strongly promotes the polarization of TAMs in alternative activated macrophages [65,66]. In turn, M2-like macrophages modify their pathways and the cytokines secreted, reversing their function and assisting the tumor’s progression. M2-like TAMs also support neo-angiogenesis in the TME by the secretion of different mediators, such as VEGF and IL-8, that are further exacerbated by the hypoxic environment [67,68]. In these cells the characteristic iNOS pathway activated from M1-macrophages is commonly replaced by several alternative pathways that lead to the production of polyamines and ornithines, as well as different cytokines such as the epidermal growth factor (EGF), PDGF, etc., that massively stimulate cancer cell proliferation [69]. M2-like TAMs are also strongly correlated to cancer metastasis production by the release of several extracellular matrix (ECM) degradation enzymes that hydrolyze the collagen fibers of the tumor-surrounding environment, promoting the spreading of metastatic cancer cells [70].

In this scenario, different approaches have been proposed to reduce or re-educate the M2-like macrophages in the TME. In particular, drug delivery systems have been widely implicated to achieve this goal, thanks to their suitable versatility both in terms of delivered bioactives and the surface modification architecture [71,72,73]. Moreover, a relatively recent, but ever growing interest is focused on the macrophage-derived EVs as both tumor markers and to realize personalized targeted nanomedicines able to restore the anticancer effect of TAMs [74,75,76].

## 3. Extracellular Vesicles from Cellular Communication Mediators to Promising Nanomedicine

EVs consist of a heterogeneous lipid-based particles’ population which includes microvesicles (MVs), exosomes and apoptotic bodies, differing each other for size, biogenesis, function pathways and delivered cargo [77]. 

Since their discovery over three decades ago [78,79], EVs’ main deepened function within cellular biology has been intercellular communication. They participate actively in cell–cell and organ–cell signaling by delivering bioactive cargos (lipids, proteins and nucleic acids) from the native donor cell to the recipient ones [80]. 

EVs are released from cells, and their presence was found in several biological fluids: human breast milk, saliva, cerebrospinal fluid and urine [81,82,83,84]. For this reason, different purification and isolation methods have been developed based on the EVs’ starting source (Table 1), as well as for the detection of their surface biomarkers and cargos. 

**Figure 2:**
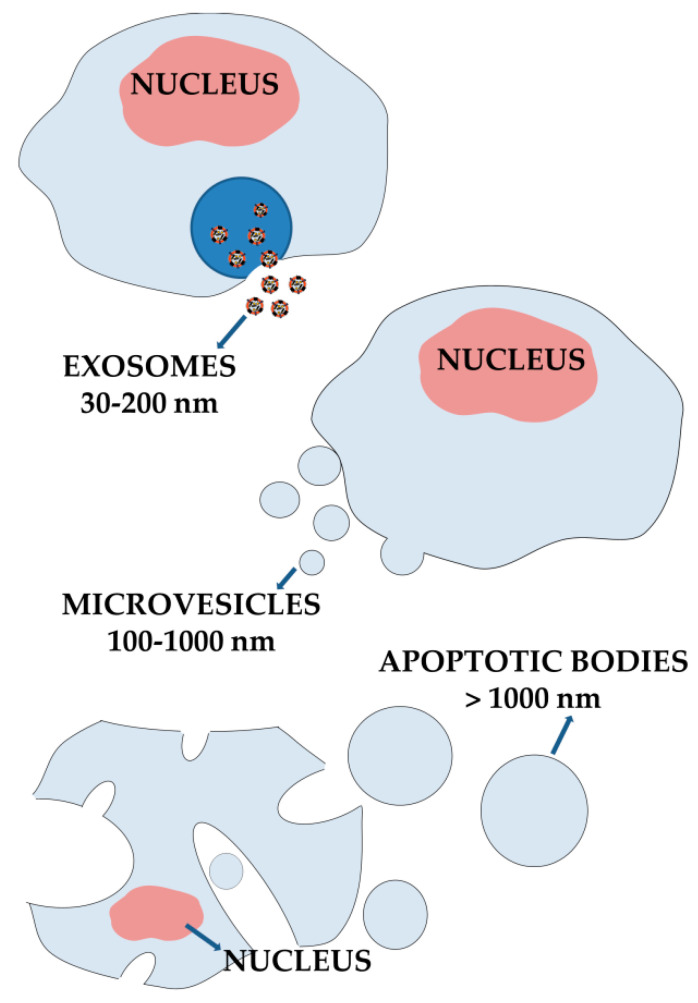
EVs’ classification and schematic representation of main biogenesis mechanisms.

Although the phospholipid bilayer supramolecular structure of EVs, a first immediate classification based on size can be carried out: MVs show a dimensional range between 100 and 1000 nm [95], exosomes between 30 and 200 nm [96] and apoptotic bodies up to several micrometers [97] (Figure 2).

EVs take origin from different biogenesis processes, including both the multi-step derivation from the endosomal plasma membrane and the direct origin from the cell plasma one [98]. Apoptotic bodies are the result of apoptotic processes, that leading to cellular components’ deterioration, then give rise to these microstructures, which are deleted through the phagocytosis’ mechanisms [99,100]. MVs’ formation, which is supposed to be the result of the budding and fission of the cell plasma membrane, is mainly due to the membrane phospholipid rearrangement mediated by aminophospholipid translocases, able to transfer the bilayer’s lipids from the inner layer to the outer one and/or vice versa, after different stimuli. This phenomenon leads to a momentary loss of the physiological charge distribution of the phospholipid bilayer, and the resulting contraction of the actomyosin cytoskeleton provides the MVs’ release [101,102,103]. Exosomes are the most investigated vesicles among EVs; their biogenesis mechanism consists of different steps: early and late endosome formation, intraluminal vesicles’ (ILVs) development and their fusion with the plasmatic membrane. All these steps are mediated by a set of several proteins known as the endosomal sorting complex required for transport (ESCRT) that collaborate for the entire process up to the exosomes’ formation [104]. Nevertheless, the role of the ESCRT was debated and the presence of this complex appears unnecessary, as suggested by Wei and co-workers who investigated the Rab GTPase, a small GTPases proteins’ group. In particular, through several analyses, Rab31 results in an increase of ILVs’ formation and exosomes’ release, thus providing an alternative ESCRT-independent biogenesis pathway [105]. Moreover, several studies also highlight that tetraspanins such as CD9, CD82, CD63 as well as the small integral membrane proteins of the lysosome/late endosome are involved in the formation process [106] and in the modulation of EVs’ membrane curvature. In these attempts, recently, Umeda and co-workers demonstrated the crystal structure of CD9, showing a reversed cone-like architecture able to induce a membrane curvature in a lipid layer, thus further confirming their role as a membrane remodeling agent and explaining their massive presence in membrane domains with a high curvature [107]. 

Apart from their physiological role as cellular mediators, the potential use of EVs as a nanoplatform for the development of advanced nanomedicine has gained ever-increasing attention in the scientific community thanks to the biocompatibility of these vesicles, as well as their natural cargo and their potential ability to induce several pathways’ activation/modification in recipient cells [108,109].

The field of oncology was ameliorated by the development of nanomedicines; in fact, the ever-increasing mortality rate, the therapies’ lack of efficacy as well as their several side effects led to the need of realizing targeted/personalized therapies, which nanomedicine is well suited to. Several strategies have been carried out in order to obtain anticancer drug delivery systems which are able to reach the tumor site and release the payloads [110]. EVs naturally fit with this purpose because of their sub-micrometer size, their origin, which confers biocompatibility, and their native surface decoration that lets them have a long circulation and targeting features [111]. The intrinsic targeting properties were recently demonstrated by Guo et al. through the use of U87 and U251 glioblastoma cell-derived EVs for the delivery of Doxorubicin [112]. In detail, during this investigation, the authors eliminated the natural cargo of isolated EVs by the use of saponine, maintaining the targeting features as unaltered. Conversely, the treatment with this surfactant abrogated the pro-tumorigenic properties of glioblastoma-derived EVs, and through their ability to cross the blood–brain barrier accumulating in the tumor microenvironment, this nanoplatform was used to encapsulate doxorubicin, in order to provide an effective targeted nanomedicine for the treatment of glioblastoma [112].

The natural surface architecture of EVs also leads to better internalization rates compared to synthetic nanoparticles [113], and so many uptake mechanisms have been clarified [114]. Among these latest, the direct fusion with the plasma membrane of the recipient cells is the most immediate surely. This event occurs because of the presence of the specific surface proteins of the recipient cell that can interact with the related receptors of the EVs and/or vice versa, thus providing the binding and fusion [115]. However, endocytosis is the main active mechanism exploited for cargo internalization, and can be mediated by different proteins and further divided into phagocytosis for micrometric particles, pinocytosis and micropinocytosis [116,117,118]. Importantly, the studies about nucleoplasmic reticulum-associated late endosomes for the intranuclear transport of EVs’ cargo, mediated by several proteins or complexes, led to new potential targeted anticancer therapeutic strategies [119].

EVs represent also a valid alternative to the conventional drug delivery systems because of the possibility to be loaded through engineering cell methods, by which chemotherapeutic drugs were packaged into EVs, as a consequence of the pre-treatment of the parent cells [120] and/or post-collection ones by the use of different techniques [121,122,123,124].

Among different donor cells, immune system cells have been particularly focused because of their native tropism towards tumor and inflammation sites. On these bases, despite sharing the same behavior with cancer cell-derived EVs, immune cell-derived ones also provide the great advantage of being deprived of the pro-oncogenic cargo [125].

## 4. Macrophage-Derived EVs for Cancer Treatment

Circulating monocytes are characterized by a natural tropism toward tumors and inflamed tissues. The high affinity of these immune cells toward these pathological environments is due to the over-expression of ICAM-1 proteins on the surface of an inflamed endothelium and the occurring interactions with several adhesion molecules on the surface of the leukocytes, e.g., lymphocyte function-associated antigen 1 (LFA-1), P-selectin glycoprotein ligand-1 (PSGL-1) and macrophage-1 antigen (Mac-1). This specific binding results in the strong adhesion of these cells to the inflamed endothelium, thus allowing their extravasation [126,127].

In this scenario, different drug delivery systems containing immune cell-derived surface proteins mediating adhesion processes have been developed, in order to improve the targeting properties of the resulting nanosystems toward the TME. For this purpose, a fascinating approach has been proposed by Molinaro et al., through the development of lipid-based nanosystems incorporating leukocytes-derived plasma membrane proteins, leading to the realization of hybrid nanovesicles called “Leukosomes” [128]. These innovative nanocarriers showed physicochemical properties comparable to the conventional liposomes, with an impressive improved affinity toward inflamed vasculature, showing a five- to eight-fold enhanced accumulation rate and a more sustained extravasation process toward perivascular sites during the 24 h period after the injection. The presence of leukocytes-derived plasma membrane proteins also led to a higher biocompatibility of the resulting hybrid nanosystems, displaying an increased evasion of the mononuclear phagocytic system (MPS) and a five-fold improved circulation extent compared to conventional liposomes [128]. The huge capability of this nanosystem to target the inflamed vasculature was exploited by the authors in order to increase the effective delivery of loaded doxorubicin in breast and melanoma cancers [127]. Likewise, in the inflammation model, leukosomes demonstrated a higher accumulation rate in the TME than liposomes (up to a nine-fold increase) and a reduced uptake by the spleen and liver. Based on these results, the anticancer efficacy of dox-loaded leukosomes were investigated in vivo on both tumor models, extending the median survival rate of 21% in triple-negative breast cancer and 62% in melanoma, compared to the free drug [127].

**Figure 3:**
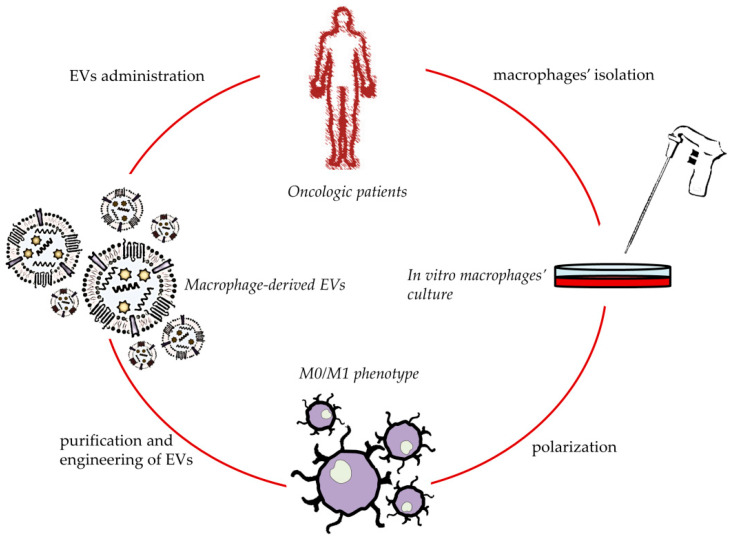
Schematic development of personalized anticancer therapies based on macrophage-derived EVs.

Based on the tumor targeting properties showed by the macrophages’ surface proteins, as well as their physiological presence in the architecture of derived EVs, an emerging tool in drug delivery is based on the use of these nanovesicles to realize anticancer targeted nanomedicines. Furthermore, taking into account their natural cargo and their role in different stages of cancer development, their implication may provide an effective anticancer immunotherapy [129] (Figure 3). 

In fact, thanks to their intrinsic ability to cross natural barriers in the body, macrophage-derived EVs can specifically deliver their payloads to hard-reachable sites, such as central nervous systems and tumor sites, reducing, at the same time, the side effects on healthy tissues [130,131]. In these attempts, recently, Haney and co-workers exploited the natural tropism of macrophage-derived EVs onto the cancer microenvironment to improve the delivery of Paclitaxel (PTX) and Doxorubicin (DOX) in triple-negative breast cancer (TNBC) [131]. During this study the authors isolated EVs from RAW 264.7 macrophages and encapsulated the two chemotherapeutics by different strategies, i.e., passive incubation, sonication or by supplementing the cells with a chemotherapeutic agent during their growth (this latest approach was tested only for DOX). Sonication statistically improved the amount of loaded chemotherapeutic agents for both drugs compared to the passive incubation. Moreover, a considerable amount of DOX was also encapsulated in the EVs by supplementing the cell growth medium with the drug, although the drug loading degree was always lower compared to the sonication approach. PEGylated liposomes loaded with the same chemotherapeutic agents were used as the control through the study. As expected, an in vitro test on the TNBC cell line (MDA-MB-231 cell line) showed that EVs were taken up on a massive scale, better than liposomes, leading to a huge increment of intracellular drug accumulation when delivered through bio-derived nanovesicles (150-fold higher for DOX and 90-times higher for PTX). A similar trend was observed after intraperitoneal (ip) and intravenous (iv) injection in an immunocompetent orthotropic TNBC mice model, showing a higher ability of macrophage-derived EVs to target the TME compared to liposomes, in response to both chemotaxis and efficient immunosystem evasion. The tested EVs also showed a specific interaction with cancer cells, reaching an EVs-mCherry-TT1 cells co-localization of ≈64% and 48% after iv and ip injection, respectively. The high tropism toward the TME also resulted in improved therapeutic properties of drug-loaded EVs compared to free drugs in vivo. Moreover, although the tumor inhibition index demonstrated by DOX-EVs was comparable to the commercially available nanoformulation Doxil^®^, the higher specificity toward the target sites may strongly reduce the side effects, thus enhancing the patients’ compliance [131]. Interestingly, the same research team demonstrated the ability of RAW 264.7 macrophage-derived EVs loaded with PTX to reverse the multidrug resistance phenomenon in P-glycoprotein (Pgp)-positive MDCK cells, providing an anticancer efficacy of a payload 50-fold higher compared to free drugs [124]. The specific mechanism that led to the higher efficacy of this formulation was not completely elucidated by the authors that demonstrated the absence of the Pgp-inhibition effect by empty EVs. In order to investigate the in vivo efficacy of this nanoformulation, the authors realized a metastatic pulmonary animal model by injecting 3LL-M27 cells in mice, who then overexpress the multidrug resistance 1 gene and then high levels of Pgp. This investigation demonstrated a proper targeting ability of macrophage-derived EVs onto lung cancer metastasis in Lewis lung carcinoma, showing a co-localization of EVs and lung metastasis of 97%. As a consequence of this high selectivity, the injection of PTX-loaded EVs significantly inhibited the metastasis growth compared to taxol, leading to around one-third of the metastasis level after treatment with bio-derived therapeutic nanovesicles compared to free drug [124].

As discussed above, the EVs surface architecture, as well as the cargo, reflects the parent native cells, and for these reasons, the macrophage polarization may be very useful in order to provide nanovesicles with the same targeting properties and intrinsic tumor-suppressive features. Indeed, the isolation of EVs from M1-like macrophages could represent a promising approach to boost the efficacy of EVs in cancer therapy [132]. In this regard, Zhao et al. recently isolated EVs from human TPH-1 macrophages after their polarization in an M1-like phenotype to provide a nanoplatform for a suitable delivery in pancreatic cancer [133]. Gemcitabine (the first-line drug in pancreatic cancer) and Deferasirox (an iron chelator able to increase the sensitivity of cancer cells toward gemcitabine by the inhibition of ribonucleotide reductase) were co-loaded into M1-like macrophage-derived EVs (M1EVs) by electroporation and tested on a GEM-resistant PANC-1/GEM cell line. Cell viability studies showed a higher cytotoxic effect of dual-loaded M1EVs compared to the combination of free drugs (cell viability rate of 29% vs. 55%, respectively). The authors attributed the increased efficacy of dual-loaded M1EVs on PANC-1/GEM to the combination of different factors, i.e., the higher activation of the hENT1 nucleoside transporter and the high calcein AM retention level of cells after treatment with therapeutic M1EVs (80% compared to the positive control Pgp inhibitor Verapamil). Furthermore, in a 3D spheroids model, the treatment with therapeutic M1EVs strongly reduces the tumor spheroids’ volume compared to the combination of free drugs after seven days (tumor spheroid volume of ≈20% and 47%, respectively), thus confirming the improved therapeutic efficiency of drugs through by M1EVs delivery [133]. 

## 5. Macrophage-Derived EVs for In Situ TAMs Phenotype’s Shifting

Macrophage-derived EVs, acting as cellular mediators, affect the polarization of TAMs. For this reason, EVs derived from M1-like macrophages were recently investigated to obtain an in situ M2 to M1 repolarization [134]. It is noteworthy that Wang et al. focused their attention on the different pathways’ activation by investigating EVs derived from M1-like or M2-like macrophages, in order to evaluate the different effect of natural cargo in these nanovesicles based on parent cells’ polarization [132]. In these attempts, the authors demonstrated that the in vitro treatment of M0 macrophages with M1EVs strongly enhanced the activation of NF-KB and i-KB compared to the control, and showed the ability of M1EVs to promote a pro-inflammatory environment by RAW264.7 macrophages/murine breast cancer 4T1 cells co-culture studies. In fact, the cellular co-culture treated with these nanovesicles showed both an increased expression of pro-inflammatory cytokines, i.e., IL6, IL12 and iNOS, and an increased activation of apoptotic processes in cancer cells through the activation of Caspase 3 pathways. These results strongly emphasized the intrinsic ability of M1-like macrophage-derived EVs to potentiate the efficacy of chemotherapeutics thanks to the creation of a pro-inflammatory microenvironment. Based on this evidence, the authors realized PTX-loaded M1EVs, in order to confirm their assumptions. The cytotoxic effect of the resulting therapeutic EVs was tested on different breast cancer cell lines, confirming the superior anticancer efficacy of PTX-loaded M1EVs compared to free PTX and PTX-loaded M2EVs (the delivery of PTX through M1EVs reduced the PTX IC_50_ around four times compared to the free drug on 4T1 cells). In vitro data were further confirmed by in vivo studies, showing a significant reduction of tumor volume and an increased survival rate after treatment with PTX-loaded M1EVs compared to free drugs. Moreover, histological analysis revealed the infiltration of M1Evs in the tumor, thus suggesting the potential ability to promote the in situ M2 to M1 transition of TAMs [132]. The ability of M1EVs to repolarize TAMs from the M2 to M1-like phenotype was recently deeply investigated in vitro and in vivo by Choo et al. in order to provide an efficacious immunotherapy able to enhance the antitumoral effectiveness of immune checkpoint inhibitors [135]. In this study the authors realized “exosomes mimetic nanovesicles” derived from M1-like macrophages (M1NVs) and obtained through the sequential extrusion of classical activated RAW 264.7 murine macrophages. The resulting M1NVs were then used to potentiate the efficacy of a clinically approved anti-PD-L1 antibody (aPD-L1) immune checkpoint inhibitor. The obtained M1NVs showed a higher enrichment of M1-markers and pro-inflammatory cytokines compared to the exosomes mimetic nanovesicles derived from M0 macrophages. The resulting M1NVs showed an in vitro higher uptake by the M2 macrophage compared to CT26 colon carcinoma cells (80.4% vs. 12%, respectively), demonstrating a higher affinity of these bio-derived nanovesicles toward macrophages. No cytotoxic effect was demonstrated after treatment on both investigated cells, showing an unaltered tumorigenesis of CT26 cells that owned a similar expression of PD-L1 mRNA after treatment with M1NVs or M0NVs. On the other hand, in vitro studies clearly demonstrated the ability of M1NVs to reverse the phenotype of the alternative activated macrophages to being M1-like. It is of note that M1NVs showed a higher macrophage-reversing phenotype ability than M1-macrophages per se (when this latest was co-cultured with M2-macrophages), probably due to the mutual influence between the two macrophages’ phenotypes in a co-culture system. The ability of M1NVs to reverse the M2-polarization of TAMs was also in vivo confirmed by using a CT26-bearing BALB/c mice model. In particular, the authors demonstrated that the maximum therapeutic efficacy was obtained through the combination of M1NVs and aPD-L1. This effect was attributed to the synergistic effect of the concomitant activation of T-cell (by the aPD-L1) and TAMs repolarization in M1-like macrophages (due to M1NVs’ natural cargos) that provided a proper antitumor effect by eliciting a Th1 cell immune response [135]. The natural tropism towards the TME and the ability of M1 macrophage-derived EVs was also exploited by Wang et al. in order to develop a targeted nanomedicine for the treatment of glioblastoma [136]. The authors provide “pre- and post-modification” in macrophage-derived EVs in order to obtain multi-responsive nanosystems able to provide a synergistic effect after in vivo administration. Briefly, after the polarization of macrophages into M1-like phenotypes, these cells were treated with a non-toxic prodrug banoxantrone (AQ4N), thus leading to the production of AQ4N-loaded M1EVs. After isolation, two hydrophobic compounds, i.e., bis(2,4,5-trichloro-6-carbopentoxyphenyl) oxalate (CPPO) and Chlorin e6 (Ce6), were embedded in the lipid bilayer of M1EVs, thus obtaining multi-stage responsive nanovesicles. Indeed, the resulting multi-loaded nanosystem showed the ability to overcome the blood–brain barrier, thus accumulating themselves in the TME as well as inducing the in situ M2 to M1 TAMs repolarization. This transition increased the tumor-suppressive features of macrophages, which further improved the production of H_2_O_2_ in the TME. In this scenario, the oxygen peroxide produced reacted with CPPO, resulting in a large amount of energy that, by activating Ce6, led to the production of a massive amount of cytotoxic reactive oxygen species (ROS). Moreover, this oxygen-consuming reaction exacerbated the hypoxia of the TME, so the M1EVs-loaded prodrug was converted into the active chemotherapeutic agent AQ4. The synergistic activity of this multi-responsive nanosystem was successfully tested on a 3D in vitro model and an in vivo one on both cell-derived xenograft and patient-derived xenograft models, demonstrating a potent anticancer effect and safe profiles on healthy tissues [136].

The herein described investigations clearly point the attention onto the multiple advantages in the use of macrophage-derived EVs compared to conventional drug delivery systems for anticancer therapies. In particular, the main gains achievable by their implication are related to their high tumor-targeting properties and the possibility to modify their cargos through the parent cells’ polarization, thus leading to a potential targeted personalized nanomedicine able to provide huge anticancer effects per se, as well as to potentiate the conventional treatment currently used in clinic.

## 6. Engineered Macrophage-Derived EVs for Anticancer Application

In order to improve the cargo-delivering capability as well as the tumor-targeting properties of EVs, several technological approaches have been investigated [137]. The main strategies to achieve this goal are based on the surface functionalization of EVs with different targeting molecules to improve the specificity toward cancer cells and/or their hybridization with conventional synthetic nanosystems, e.g., liposomes, to provide peculiar physicochemical properties to the resulting hybrid vesicles, such as physical stimuli responsiveness [138,139,140]. However, the use of these approaches, in particular, the modification of the EVs’ surface properties, still remains controversial because of their potential capability to affect the biocompatibility of these nanovesicles and/or facilitate their uptake from RES, thus speeding up their clearance. 

Despite the natural tropism of macrophage-derived EVs for the TME, several investigations have explored the potential surface’s modifications of these nanovesicles to further enhance their tumor targeting properties. For example, Kim and co-workers modified the surface of PTX-loaded RAW 264.7 macrophage-derived vesicles with aminoethyl anisamide-polyethylene glycol (AA-PEG) moiety in order to target cancer cells that overexpressed the sigma receptor [141]. This surface modification led to an increased in vitro uptake of functionalized EVs in lung carcinoma cells (3LL-M27), compared to the unmodified vesicles (over double). Interestingly, the PEGylation of EVs in the absence of the AA ligand inhibited the uptake of resulting nanovesicles, due to the hampered interaction between the cells and surface proteins of macrophage-derived EVs, thus underlining their pivotal role in assisting the cellular uptake process. The implication of sigma receptors in the cellular internalization process was confirmed by using competitive binding with free AA-PEG moiety, while the crucial role of the macrophage-derived EVs’ surface in the interaction with investigated cancer cells was further confirmed by digestion with proteinase K. In fact, this assay unambiguously showed that the removal of the EVs’ surface proteins strongly reduced their resulting cellular internalization. Moreover, in vivo studies confirmed the data obtained in vitro, showing a higher co-localization of functionalized EVs and lung metastasis compared to naïve EVs i.v. injected (≈94.4% vs. 21.8%, respectively). Based on these results, the authors investigated the antineoplastic efficacy of different PTX-loaded formulations, showing a more effective lung metastasis eradication of PTX-functionalized EVs compared to PTX-naïve EVs and free drugs [141]. A similar approach has been proposed by Li and co-workers through the realization of RAW 264.7 macrophage-derived EVs hybridized by DOX-loaded PLGA nanoparticles and the surface functionalized by the conjugation of small peptides able to bind the mesenchymal–epithelial transition factor (c-Met) to the target TNBC cells [142]. In vitro analysis on MDA-MB-231 demonstrated the crucial role of the macrophage-derived EVs’ surface proteins, showing an intracellular DOX accumulation 3.31 times higher in cells treated with PLGA-EVs DOX-loaded hybrid nanosystems than DOX-loaded PLGA nanoparticles after 4 h of incubation. Moreover, the surface functionalization of the hybrid nanosystem with the c-Met targeting peptide further increased the uptake ratio, resulting in a DOX accumulation almost double compared to unconjugated hybrid nanosystems. Obviously, the higher uptake of peptide conjugated hybrid nanosystems led to the highest apoptosis rate after 12 h of incubation, which was specifically 39.73%, 29.27%, 11.33% and 10.58% for the targeted hybrid system, non-functionalized hybrid system, PLGA nanoparticles and free DOX, respectively. In vivo studies confirmed the data obtained in vitro, demonstrating higher tumor-targeting properties of functionalized hybrid nanosystems compared to the unconjugated ones and PLGA nanoparticles (1.62 and 2.22-fold higher, respectively). It is noteworthy that the unconjugated hybrid nanovesicles showed higher tumor-targeting properties compared to PLGA nanoparticles, highlighting and further confirming in vivo the crucial role of macrophage-derived surface proteins showed in vitro. As a consequence of the different accumulation rates of the investigated formulations, the in vivo anticancer effect of the DOX-loaded formulations was strongly affected by the surface architecture, as reported by the following: conjugated hybrid nanosystems > unconjugated hybrid nanosystems > PLGA nanoparticles [142]. The surface functionalization of macrophages’ (THP-1)-derived EVs to improve the delivery of DOX in TNBC was also investigated by Gong et al. [143]. In particular, in this study, the authors provided the functionalization of EVs by stimulating THP-1 cells with phorbol 12-myristate 13-acetate (PMA), leading to the overexpression of disintegrin and metalloproteinase 15 (A15) on the surface of derived EVs. In particular, this protein contains an RGD motif in its structure which enhances the interaction with α_v_β_3_ integrin, which is overexpressed in several tumors [144]. The resulting functionalized EVs were then used to co-deliver DOX and Cho-miR159 to provide a synergistic anticancer therapy. The higher uptake rate of A15-EVs on TNBC cell lines was confirmed in vitro, showing an uptake rate of 78.6% and 15.23% for A15-EVs and unmodified EVs (n-EVs), respectively, on MDA-MB-231, and an uptake extent of 89.76% and 24.13% for A15-EVs and n-EVs, respectively, on the B16 cell line. Moreover, no significant differences were observed between the two formulations on MCF-7 cells (that express a little amount of α_v_β_3_), thus confirming that the increased uptake of A15-EVs in MDA-MB-231 and B16 cells occurred via the interaction of A15 and α_v_β_3_ integrin. The high uptake ratio of A15-functionalized EVs also provided an intracellular accumulation of payloads (DOX and Cho-miR159), increasing the apoptotic effect of Cho-miR159 compared to the free compound (47.15% vs. 28.26%, respectively) and showing a synergistic anti-proliferative effect of DOX and Cho-miR159 in MDA-MB-231. The synergistic effect of payloads, in vivo tested, provided a high tumor-targeting property of the resulting dual-loaded A15-EVs and a tumor inhibition rate of 92.8%, that was significantly higher compared to free drugs and single-loaded A15-EVs [143]. Another fascinating approach to improve the efficacy of current therapies in triple-negative breast cancer was recently proposed by Li et al. [145]. Briefly, the authors incubated the RAW 264.7 macrophages with several compounds, i.e., DOX, 5-aminolevulenic acid (converted by cells in photosensitizer PpIX) and DSPE-PEG folate, in order to obtain non-genetically engineered EVs for a potential targeted photo-chemotherapy in breast cancer. Moreover, this approach allowed the avoidance of the modification of the EVs after their release from macrophages (i.e., drug loading and/or post-conjugation approach) that may result in an excessive perturbation of the EVs’ membrane integrity. This approach led to an increased drug loading efficacy and an improved in vitro accumulation of “bio-packed DOX-PpIX-dual loaded FA-functionalized EVs” compared to “external-loaded EVs”. Moreover, this approach also contributed to the dendritic cells’ maturation (49.3% and 31.0% for bio-synthetized EVs and external-loaded EVs, respectively), showing an enhanced immunostimulation of bio-packed FA-EVs. The higher targeting properties of FA-modified EVs were in vivo confirmed on BALB/c mice orthotropic-bearing 4T1 models, showing a two-fold higher tumor accumulation rate than EVs without FA (n-EVs). The highest accumulation rate also reflected the better outcomes of the photo-chemotherapy of dual-loaded FA-EVs than co-loaded n-EVs, demonstrating an increasing tumor size ratio of 0.90 and 2.2, respectively, after 20 days [145].

Apart from the surface modification and direct loading procedures used to post-modify EVs, another approach consists in the realization of hybrid nanosystems made up of EVs and conventional nanocarriers, in order to maximize the advantage of both delivery systems [146]. In these attempts, recently Rayamajhi and co-workers realized J774A.1 macrophage-derived EVs–liposomes hybrid nanovesicles for the treatment of triple negative breast cancer [147]. In detail, the authors isolated the EVs from the murine macrophages culture media and homogenized them by the extrusion technique. The resulting homogeneous EVs’ suspension was then used to hydrate a dry thin lipid film (EVs protein: liposomal lipids ratio 1:5). The size distribution was reduced and homogenized by the extrusion technique, while for the therapeutic hybrid nanovesicles, DOX was loaded during the hydration stages. In vitro studies showed a three and four times higher uptake ratio of hybrid nanosystem by the TNBC 4T1 cell line and K7M2 osteosarcoma, respectively, compared to liposomes. The authors described the increased uptake ratio to the presence of transmembrane macrophage-derived proteins on the surface of hybrid nanovesicles that were not affected by hybridization procedures. The increased interaction rate also strongly reduced the IC50 value of DOX compared to the free drug for both cell lines. Conversely, no significant differences were observed between the hybrid nanosystem and liposomes in terms of internalization into the murine fibroblast NIH/3T3, demonstrating a natural tropism of these nanovesicles toward tumor cells [147].

All the strategies described have demonstrated a different technological approach to engineer macrophage-derived EVs in order to further improve their potential application in cancer therapies. As specified, these nanovesicles lend themselves well to several modification processes, thus showing a great versatility. Among the several paths investigated to date, it is our opinion that the “pre-engineering approaches” through the modification of parent cells in order to induce specific features to the released EVs and the hybridization of EVs with conventional drug delivery systems are the most promising ones. In particular, this latest may strongly improve the features of naïve EVs, thus leading to the realization of “next-generation” nanomedicine able to exploit the natural properties of EVs, showing, at the same time, the peculiar physicochemical characteristics provided by synthetic nanocarriers, such as stimuli responsiveness.

## 7. Conclusions and Future Perspectives

Cancer affects all ages of the worldwide population, but to date, no effective therapies have been made commercially available. In this context, the scientific world started to explore all the possible approaches in order to realize a safe and efficacious treatment. Recently, nanotechnology and biology have reached promising results in the extracellular vesicles field. EVs are the main class of the communication mediators into the body by delivering several bioactives such as lipids, RNA, DNA and proteins. They are involved in many physiological and pathological pathways, such as cancer. In particular, due to the pivotal role played by immune cells in the TME, macrophage-derived extracellular vesicles are a subclass of EVs which are able to exploit all the features of the donor cells, such as the tropism towards inflamed tissue and tumor sites, a typical macrophagic characteristic. Among the macrophagic phenotypes, M1 macrophages as a source for EVs fit with the purpose, let the derived EVs reflect their oncosuppressive and proinflammatory capability and are useful for the realization of a personalized effective anticancer therapy. These nanovesicles, including hybrid ones, may then provide a suitable nanoplatform as a starting point for the development of “new-generation” anticancer nanomedicines, based on different patients’ genetic and genomic profiles. In particular, the opportunity to isolate macrophages directly from the patients’ blood and then obtain EVs that can be properly modified in a laboratory may lead to the development of effective personalized nanomedicines. This promising approach may then improve both the biocompatibility and the efficacy of the current anticancer treatments, thus guaranteeing high standard goals and a better patients’ compliance.

## Figures and Tables

**Table 1:** Main extracellular vesicles’ isolation and purification methods.

Method	Functional Principle	Main Advantages/Disadvantages	References
Differential centrifugation	Multi step precipitation	Cheap method/low EVs recovery rate and unspecific method	[85,86]
Polymer-based precipitation	Salting out method	Fast method/unspecific method and low purity	[87,88]
Size-exclusion chromatography	Hydrodynamic radius-based separation	High purity of EVs recovered/ low yield	[89,90]
Tangential flow filtration (TFF)	Cross-flow filtration	Large scale isolation/unspecific method	[91]/NA
Magnetic beads affinity	Immunocapture	Efficient multi-step method/expensive and unsuitable for large sample volumes	[92,93]
Microfluidic platforms	Immunocapture	Single-step method/Shear stress can damage EVs	[93,94]

## Data Availability

Not applicable.
